# Uremic Toxins in the Progression of Chronic Kidney Disease and Cardiovascular Disease: Mechanisms and Therapeutic Targets

**DOI:** 10.3390/toxins13020142

**Published:** 2021-02-13

**Authors:** Yong Jin Lim, Nicole A. Sidor, Nicholas C. Tonial, Adrian Che, Bradley L. Urquhart

**Affiliations:** 1 Department of Physiology and Pharmacology, Schulich School of Medicine and Dentistry, The University of Western Ontario, London, ON N6A 3K7, Canada; ylim33@uwo.ca (Y.J.L.); nsidor@uwo.ca (N.A.S.); ntonial@uwo.ca (N.C.T.); ache3@uwo.ca (A.C.); 2 Division of Nephrology, Department of Medicine, Schulich School of Medicine and Dentistry, The University of Western Ontario, London, ON N6A 3K7, Canada; 3 Lawson Health Research Institute, London, ON N6A 3K7, Canada

**Keywords:** uremic toxins, chronic kidney disease, cardiovascular disease, indoxyl sulfate, p-cresyl sulfate, hippuric acid, trimethylamine N-oxide, asymmetric dimethylarginine, tumor necrosis factor al-pha, interleukin 6

## Abstract

Chronic kidney disease (CKD) is a progressive loss of renal function. The gradual decline in kidney function leads to an accumulation of toxins normally cleared by the kidneys, resulting in uremia. Uremic toxins are classified into three categories: free water-soluble low-molecular-weight solutes, protein-bound solutes, and middle molecules. CKD patients have increased risk of developing cardiovascular disease (CVD), due to an assortment of CKD-specific risk factors. The accumulation of uremic toxins in the circulation and in tissues is associated with the progression of CKD and its co-morbidities, including CVD. Although numerous uremic toxins have been identified to date and many of them are believed to play a role in the progression of CKD and CVD, very few toxins have been extensively studied. The pathophysiological mechanisms of uremic toxins must be investigated further for a better understanding of their roles in disease progression and to develop therapeutic interventions against uremic toxicity. This review discusses the renal and cardiovascular toxicity of uremic toxins indoxyl sulfate, p-cresyl sulfate, hippuric acid, TMAO, ADMA, TNF-α, and IL-6. A focus is also placed on potential therapeutic targets against uremic toxicity.

## 1. Introduction

### 1.1. Chronic Kidney Disease

Chronic kidney disease (CKD) is defined as abnormalities of kidney structure or function present for more than three months [1]. Globally, CKD is estimated to affect 8–16% of the population, with the prevalence increasing with age [1,2]. The Kidney Disease: Improving Global Outcomes (KDIGO) guidelines classifies CKD into five categories based on glomerular filtration rate (GFR) and three categories based on level of albuminuria, where GFR <60 mL/min/1.73 m^2^, albuminuria >30 mg/24 h, or presence of other markers of kidney damage (i.e., electrolyte abnormalities or histological/structural abnormalities) for longer than three months indicates CKD [3]. The loss of kidney function in CKD is progressive and irreversible. Renal replacement therapies, including dialysis and kidney transplantation, are necessary for patients who progress into end stage renal disease (ESRD; GFR <15 mL/min/1.73 m^2^). Late-stage CKD patients have a 3.6-fold increased risk for mortality compared to the general population, with risk being further increased to 9- to 12-fold for patients receiving dialysis treatment [4]. The life expectancy of a 55-year-old patient with stage 5 CKD or ESRD is only 5.6 years, worse than some forms of cancer [5].

### 1.2. Chronic Kidney Disease and Cardiovascular Disease

It is well established that CKD patients have increased risk for cardiovascular disease (CVD), with increasing incidence of cardiovascular events as CKD progresses [6]. Although the traditional risk factors for CVD such as hypertension, advanced age, dyslipidemia, and diabetes mellitus are prevalent in CKD, CKD patients are faced with additional “non-traditional” CKD-specific risk factors such as volume overload, malnutrition, anemia, oxidative stress and inflammation [7]. Cardiovascular complications such as left ventricular abnormalities, cardiomyopathy, atherosclerosis, and vascular calcification are more prevalent and/or accelerated in CKD patients as a result of decreased kidney function [7]. Consequently, CVD significantly increases the risk for morbidity and mortality in CKD patients and is the most common cause of death in patients with CKD [8,9]. Additionally, the risk of CKD progression to ESRD increases in conjunction with the severity of hypertension; CKD patients with a baseline blood pressure ≥180/100 mmHg have an approximately 15-fold greater risk of developing ESRD compared to normotensive CKD patients [10]. 

### 1.3. Uremia

Uremia is a complication of CKD and is defined as the accumulation of solutes that are normally cleared by the kidneys [11]. If left untreated, uremia is a life-threatening condition. Though dialysis greatly prolongs the survival of ESRD patients, it is unable to completely mitigate the uremic condition, leaving patients with what is referred to as “residual syndrome” [11]. Despite regular dialysis treatment, the incomplete removal of organic waste compounds results in accumulation of uremic toxins, which play a crucial role in the progression of CKD and CVD. To date, over 100 uremic toxins have been identified and classified by The European Uremic Toxin Work Group (EUTox) [12]. The EUTox classifies uremic toxins by their physicochemical properties into the following categories: free water-soluble low-molecular-weight solutes (<500 Da), protein-bound solutes, and middle molecules (≥500 Da) [12].

## 2. Protein-Bound Uremic Toxins

Protein-bound uremic toxins constitute approximately 25% of all currently identified uremic toxins [12]. As a consequence of being highly bound to plasma proteins, protein-bound uremic toxins are poorly cleared by dialysis [13]. Many of these protein-bound uremic toxins are gut-derived and are the by-products of aromatic amino acid breakdown by intestinal bacteria.

### 2.1. Indoxyl Sulfate

**Figure 1:**
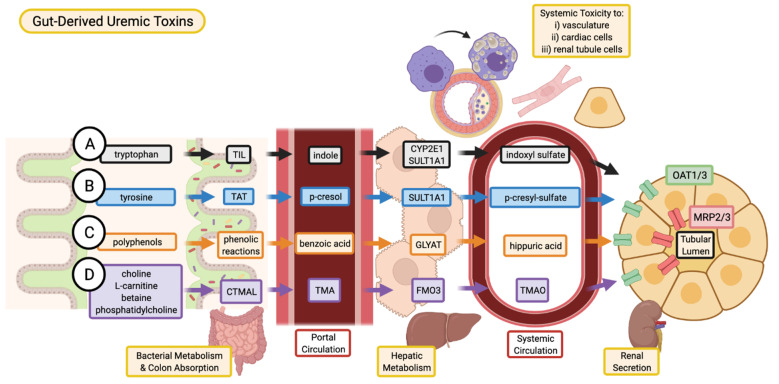
Absorption, and excretion pathway of gut-derived uremic toxins. (**A**) Indoxyl sulfate is produced when dietary tryptophan is converted into indole by bacterial tryptophan indole-lyase (TIL) and subsequent absorption into the portal circulation for further metabolism by cytochrome P450 2E1 (CYP2E1) and sulfotransferase 1A1 (SULT1A1). (**B**) P-cresyl sulfate begins as dietary tyrosine which is metabolized by tyrosine aminotransferase (TAT) into p-cresol. These intermediates are converted into p-cresyl sulfate through SULT1A1. (**C**) Various dietary polyphenols are converted through multiple phenolic reactions by colon bacteria into benzoic acid which is then conjugated with glycine by glycine-N-acyltransferase (GLYAT) to produce hippuric acid. (**D**) TMAO is the product of TMA oxidation by flavin-containing monooxygenase 3 (FMO3), where TMA is the intermediate metabolite of carnitine trimethylamine lyase (CTMAL) breakdown of various dietary molecules such as choline, L-carnitine, betaine, and phosphatidylcholine. (**A**–**D**) Uremic toxins are secreted by renal tubular cell via drug transporters (organic anion transporter 1/3, OAT1/3; and multidrug resistance-associated proteins 2/3, MRP2/3 and subsequently excreted in the urine. Image created with BioRender.com.

Indole is a metabolic product of tryptophan decomposition by bacterial tryptophanase. Once produced by intestinal bacteria, indole is absorbed into the portal circulation and enters the liver (Figure 1) [14]. In the liver, indole is hydroxylated by cytochrome P450 2E1 (CYP2E1) to form 3-hydroxy indole [15] and subsequently sulfated by sulfotransferase 1A1 (SULT1A1) to produce indoxyl sulfate (IS) [16]. IS is extensively excreted in the urine by proximal tubular secretion through basolateral organic anion transporter 1 (OAT1) and OAT3 [17]. IS is highly protein-bound (Table 1) to albumin in the circulation (93%) [18] and is consequently poorly cleared by dialysis [19]. As kidney function declines, IS levels increase in the blood and this elevation contributes to further progression of CKD [20].

#### 2.1.1. Mechanisms for the Progression of CKD

**Figure 2:**
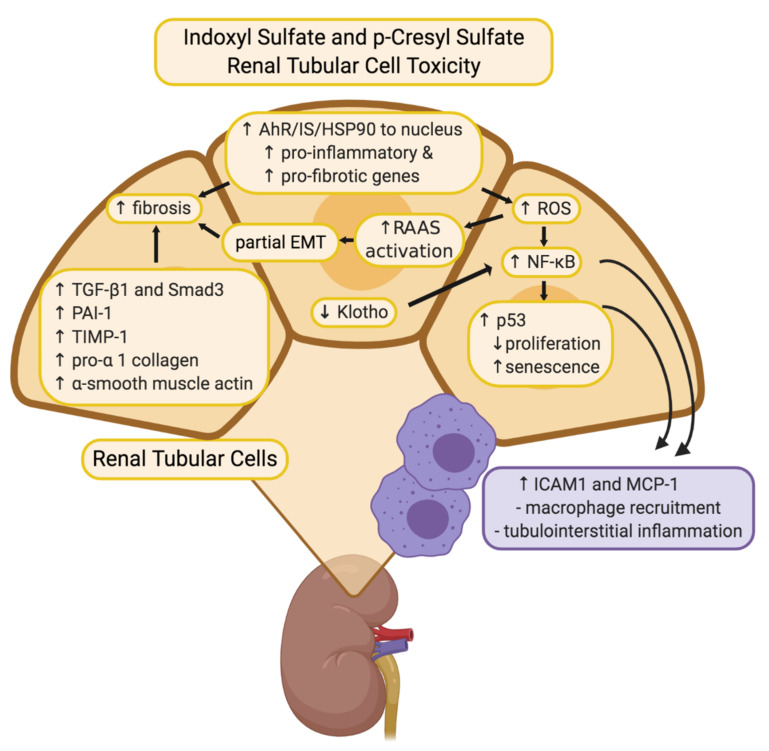
Overview of putative mechanisms of renal toxicity for IS and pCS. Through various inflammatory and fibrotic pathways, both IS and pCS have been described to mediate toxicity to renal tubular cells in similar ways. Increased expression of various fibrotic genes such as TGF-β1, TIMP-1, pro-α1 (I) collagen contribute to alterations in the tubular cell morphology and structure of the ECM. Involvement of ROS and NF-κB reduce the proliferative capacity of tubular cells, contribute to partial EMT leading to fibrosis, and recruit macrophages to produce additional tubulointerstitial inflammation. Image created with BioRender.com.

IS has shown nephrotoxic effects through generation of reactive oxygen species (ROS) [24], depletion of anti-oxidative systems [25], and induction of fibrosis and inflammation (Figure 2). NF-κB plays a central role in the pathological effects of IS in the kidneys. In human proximal tubule cells (HK-2) the activation of NF-κB by IS suppressed cellular proliferation, induced and accelerated senescence through induction of p53, and promoted fibrosis by inducing TGF-β1 and PAI-1 expression [24,26]. p53 induction was also proposed to contribute to renal fibrosis by stimulating the expression of TGF-β1 and subsequent activation of Smad3 [27]. Epithelial-mesenchymal transition (EMT) of tubular epithelial cells has long been considered a pro-fibrotic mechanism. Recent evidence indicates that renal epithelial cells undergo a partial-EMT where epithelial cells develop fibroblast-like characteristics, without a complete conversion to fibroblasts [28]. IS induced an EMT-like process in vitro and in vivo through activation of the renin-angiotensin system (RAS), further contributing to renal fibrosis [29]. Moreover, IS induces renal expression of intercellular adhesion molecule 1 (ICAM-1), which is associated with monocyte infiltration into the kidney [30], and monocyte chemoattractant protein (MCP-1), a chemotactic cytokine implicated in macrophage recruitment and activation in tubulointerstitial inflammation [31]. 

The expression of Klotho, an anti-aging gene with renoprotective properties [32], is reduced in CKD patients [33]. IS was shown induce Klotho depletion in vitro and in vivo [34], which has been linked with increased activation of NF-κB [35]. IS-induced Klotho depletion may be facilitated epigenetically through the CpG hypermethylation of the Klotho gene [36]. 

#### 2.1.2. Mechanisms for the Progression of CVD

**Figure 3:**
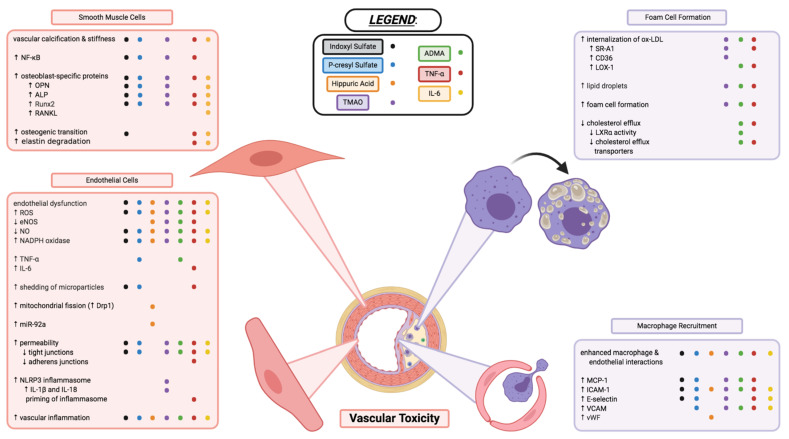
Vascular toxicity of uremic toxins through impacts on endothelial cell function, vascular smooth muscle cell morphology, and macrophage recruitment and transformation into foam cells of atherosclerotic plaques. Each coloured dot (see below and legend in figure) represents a uremic toxin having evidence in the literature to contribute to the various processes, or changes in cell activity and function described. Indoxyl sulfate (●; black), p-cresyl sulfate (●; blue), hippuric acid (●; orange), TMAO (●; purple), ADMA (●; green), TNF-α (●; red), and IL-6 (●; yellow) are shown to have varying effects in the cell types and cellular processes described above. Endothelial dysfunction and inflammation, vascular calcification and stiffness, enhanced interactions between macrophages and endothelial cells, as well as formation of foam cells are critical components to the various uremic toxins’ vascular toxicities. Some toxins may contribute to these mechanisms of toxicity but have yet to be elucidated or confirmed in the literature; some toxins have contradicting evidence that needs further investigation. This figure does not necessarily describe the complex interplay between the uremic toxins, inflammatory markers, and immune system that likely play a large interconnected role in the vascular toxicity contributing to CVD. Image created with BioRender.com.

IS is also implicated with CVD and has clinically been correlated with mortality and comorbidities such as vascular calcification, vascular stiffness, and congestive heart failure in patients with ESRD [37]. IS is proposed to have detrimental effects on both cardiac cells and the vasculature (Figure 3). In vitro studies have shown that IS may play a role in endothelial dysfunction – an early marker of atherosclerosis [37]. In human umbilical vein endothelial cells (HUVECs), IS increased ROS production, decreased nitric oxide (NO) production and cell viability, and induced mRNA expression of NADPH oxidase 4 (Nox4) [38]. IS upregulates the expression of MCP-1 [39] and cell adhesion molecules E-selectin [40] and ICAM-1 [39], enhancing leukocyte interaction with endothelial cells and facilitating inflammation.

Another common vascular complication in CKD is vascular calcification (Figure 3), which increases in prevalence as kidney function declines [41]. Vascular calcification is a risk factor for cardiovascular mortality and morbidity [41]. The differentiation of vascular smooth muscle cells (VSMCs) from a contractile to osteogenic phenotype is believed to be a key player in the development of vascular calcification in CKD. In both in vitro [42] and in vivo [43] experiments, IS has been shown to increase the expression of osteoblast-specific proteins, including runt-related transcription factor 2 (Runx2) and osteopontin (OPN). The activation of a PI3K/Akt/NF-κB pathway was shown to play a potential role in the osteogenic effects of IS [44]. 

IS also has direct pro-hypertrophic, pro-fibrotic, and pro-inflammatory effects on cardiomyocytes and cardiac fibroblasts. IS stimulated hypertrophy of neonatal rat cardiomyocytes, collagen synthesis in neonatal rat cardiac fibroblasts, and increased mRNA expression of pro-inflammatory cytokines in THP-1 cells (immortalized monocyte-like cell line), ultimately contributing to cardiac remodeling. These effects were proposed to be mediated through the activation of mitogen-activated protein kinase (MAPK) and NF-κB pathways [45]. Furthermore, cardiomyocytes treated with IS exhibited increased oxidative stress and reduced expression of UCP2, a member of the mitochondrial uncoupling proteins family with cardio-protective properties against ROS [46]. Restoring UCP2 in cardiomyocytes conferred protection against IS-induced oxidative stress and UCP2 downregulation [46].

### 2.2. p-Cresyl Sulfate

Metabolism of tyrosine and phenylalanine by intestinal bacteria yields a number of phenol derivatives, one of which is p-cresol [47]. P-cresol is absorbed and subsequently sulfated in the liver by SULT1A1 to produce p-cresyl sulfate (pCS) [48] (Figure 1). In vivo, more than 95% of p-cresol circulates as pCS [49]. Like IS, pCS is highly bound (90%) to albumin and is poorly cleared by dialysis [50] (Table 1). pCS is mainly cleared from the body by tubular secretion facilitated by basolateral uptake by OATs, namely OAT1/3 [51].

#### 2.2.1. Mechanisms for the Progression of CKD

The accumulation of pCS shows similar toxic effects to IS. Much like IS, pCS induces oxidative stress and renal fibrosis/inflammation, and clinical studies have associated pCS with CKD progression [47] (Figure 2). pCS-treated HK-2 cells showed decreased viability, increased NADPH oxidase activity, and increased mRNA expression of TGF-β1, TIMP-1, and pro-α1 (I) collagen [52]. Similarly, kidneys from nephrectomised rats had increased tubular degeneration and fibrosis, increased TGF-β1 production, increased superoxide production, and upregulation of NADPH oxidase activity and expression [52]. pCS administration to nephrectomised rats resulted in CpG hypermethylation of the Klotho gene and reduced expression of Klotho in renal tubular cells [36]. Like IS, pCS also induces an EMT-like process through the activation of the RAS pathway, and the fibrotic effects of pCS were attenuated by inhibition of the RAS pathway [29]. P-cresyl sulfate has been shown to inhibit efflux transporters MRP4 and BCRP in proximal tubule cells, which may cause intracellular accumulation of toxins, including p-cresyl sulfate itself [53]. 

#### 2.2.2. Mechanisms for the Progression of CVD

In hemodialysis patients, serum pCS levels were reported to be higher in patients with carotid atherosclerotic plaque and positively correlated with increased total plaque area. Serum pCS levels were also independently associated with the incidence and progression of carotid atherosclerotic plaque [54] and a significant independent predictor of plaque burden in patients attending vascular prevention clinics [55]. 

pCS has been implicated in vascular inflammation, vascular calcification, and atherogenesis. Cultured human endothelial and aortic smooth muscle cells treated with pCS showed enhanced ROS production, increased NADPH oxidase expression [56], and increased expression of pro-inflammatory factors MCP-1 and TNF-α [54]. pCS also increased mRNA levels of osteoblast-specific proteins in HASMCs, including alkaline phosphatase (ALP) and OPN, indicating a potential role of pCS in vascular calcification [56]. A recent study demonstrated pCS-induced ROS induces the phosphorylation of JNK, p38, and ERK, subsequently leading to increased NF-κB mediated expression of Runx2 and ALP [57]. pCS was also shown to increase the expression of adhesion molecules E-selectin, ICAM-1, and vascular cell adhesion molecule 1 (VCAM-1), promoting leukocyte-endothelium interaction in both endothelial cells and nephrectomised apoE-/- mice [54]. 

### 2.3. Targets for Therapeutic Intervention—IS and pCS

The most extensively studied method for lowering serum levels of gut-derived uremic toxins is oral administration of the spherical carbon adsorbent AST-120. AST-120 adsorbs precursors of gut-derived uremic toxins, including indole and p-cresol, in the colon and prevents their absorption into the circulation. A number of studies have demonstrated the ability of AST-120 administration to reduce plasma concentration of IS and pCS in both animal models [58] and hemodialysis patients [59]. In vivo, AST-120 has shown reno- and cardio-protective effects against uremic toxins [60,61]. Clinically, the benefits of AST-120 remain controversial. Although AST-120 has been shown to slow the decline of GFR in some studies [62], the large international randomized controlled trials EPPIC-1/EPPIC-2 showed a lack of benefit in delaying the progression of CKD, based on the primary endpoints of doubling of serum creatinine and initiation of dialysis or transplantation [63]. However, eGFR, a secondary endpoint in the EPPIC trials, declined significantly less with AST-120 treatment than placebo in EPICC-2 and in a pooled analysis of both trials. Furthermore, upon post hoc analysis of the EPPIC trials, it was suggested that AST-120 may delay CKD progression in certain patient subgroups [64,65]. While this post hoc analysis is intriguing, the best available evidence suggests AST-120 does not slow progression of CKD and further studies may be warranted in specific patient subgroups.

In addition to decreased clearance of gut-derived uremic toxins, CKD patients have a greater abundance of intestinal bacteria that produce indole and p-cresol [66]. Manipulation of the gut microbiome through the administration of pre-, pro- and synbiotics has been investigated as a therapeutic strategy for reducing the production of gut-derived uremic toxins. Overall, in healthy subjects, CKD patients, and hemodialysis patients, pre-, pro-, and synbiotics are reported to have a positive benefit in reducing the production of IS and pCS [67,68]. Currently, there are multiple registered clinical trials with plans to further investigate the effects of probiotics (NCT04390347) and synbiotics (NCT04527640) on IS and pCS levels in the setting of kidney disease. Using small molecules for targeting the gut microbiome may be another method of manipulating synthesis of gut-derived uremic toxins. Isoquercitrin, a naturally occurring small molecule, was recently demonstrated to reduce indole production without having microbicidal activity or inhibiting tryptophanase activity. It was proposed that isoquercitrin suppressed indole production by reducing tryptophan transport into bacteria through inhibition of the bacterial electron transport chain protein complex I and weakening the proton motive force [69].

By a similar rationale, modification of diet is another strategy to reduce uremic toxin production by gut bacteria. As IS and pCS are the result of bacterial breakdown of tryptophan and tyrosine/phenylalanine, respectively, reducing the dietary intake of these amino acid precursors via protein restriction can reduce the production of these gut-derived uremic solutes. In a protein restriction study, healthy subjects receiving a low protein diet had lower plasma levels and 24-h urinary excretion of IS than those receiving a high protein diet [70]. A decreasing trend in plasma levels and 24-h urinary excretion of pCS was also observed with the low protein diet, although the difference was not statistically significant (*p* = 0.07) [70]. It was also demonstrated that the dietary protein to fiber ratio was associated with both total serum IS and pCS levels in CKD patients [71]. Although evidence exists for the beneficial effects of protein restriction, protein malnutrition is common in CKD patients and may be exacerbated by dietary protein restriction [72]. Many clinical trials are planning to investigate the efficacy of dietary modulation in kidney disease (i.e., NCT03959228, NCT04505462).

### 2.4. Hippuric Acid

Hippuric acid is a gut-derived, protein-bound uremic toxin elevated in CKD [12]. The gut microbiome converts dietary polyphenols into benzoic acid through multiple phenolic reaction pathways, which is subsequently converted into hippuric acid via conjugation with glycine in the liver or kidneys [73] by glycine-N-acyltransferase (GYLAT) [74]. In circulation, hippuric acid is approximately 34% bound to albumin [75] and clearance of hippuric acid by dialysis is 64% [76]. OATs may play a role in the excretion of hippuric acid by the kidney [77]. 

#### 2.4.1. Mechanisms for the Progression of CKD

Although there is some evidence suggesting hippuric acid contributes to the progression of renal fibrosis, the mechanisms of toxicity are not as well defined as IS and pCS. Hippuric acid has been implicated in promoting renal fibrosis and endothelial dysfunction by inducing oxidative stress. A recently published study demonstrated that hippuric acid contributes to the progression of renal fibrosis by disrupting redox homeostasis [78]. In this study, incubation of HK-2 cells with hippuric acid resulted in expression of fibrosis markers and induced extracellular matrix (ECM) imbalance, increased ROS and Nox4 expression, and activated TGF-β/Smad signaling. Nuclear factor erythroid 2-related factor 2 (NRF2) is a transcription factor that regulates the expression of thiol molecules, antioxidants, and detoxifying enzymes [78]. The formation of a NRF2-KEAP1-CUL3 complex negatively regulates NRF2 by ubiquitinating it for degradation. Under oxidative stress, the NRF2-KEAP1-CUL3 complex is disrupted and NRF2 can translocate into the nucleus to drive expression of the antioxidant network. Hippuric acid was shown to decrease the expression of NRF2 and its downstream antioxidant enzymes, and increase oxidative stress. Treatment with an NRF2 activator alleviated the reductions in NRF2 and antioxidant activity, and NRF2 was therefore proposed to be a potential therapeutic target against hippuric acid induced fibrosis [78]. 

#### 2.4.2. Mechanisms for the Progression of CVD

Hippuric acid has been reported to promote endothelial dysfunction in vitro and in vivo, via generation of mitochondrial ROS. A shift in the balance between mitochondrial fusion and mitochondrial fission towards mitochondrial fission results in mitochondrial fragmentation and increased ROS generation. Treatment of human aortic endothelial cells (HAECs) with hippuric acid induced mitochondrial ROS production, reduced eNOS expression and increased expression of endothelial dysfunction markers ICAM-1 and von Willebrand factor (vWF). The mitochondria of HAECs treated with hippuric acid also exhibited morphological changes indicative of mitochondrial fragmentation and increased expression of Dynamin-related protein 1 (Drp1), a major regulator of mitochondrial fission. These effects on the endothelium were confirmed in vivo using nephrectomised CKD rats and healthy rats treated with hippuric acid. Collectively, these results suggest that hippuric acid promotes endothelial dysfunction at least partly by inducing mitochondrial fission and mitochondrial ROS production [79].

Another mechanism of hippuric acid-induced endothelial dysfunction may be through induction of miR-92a in endothelial cells. Hippuric acid was shown to induce miR-92a, a microRNA induced by oxidative stress in endothelial cells. miR-92a has been implicated in the angiogenic and atherosclerotic process and is increased in patients with CKD and pre-clinical models of CKD [80].

In a clinical study involving 80 hemodialysis patients, hippuric acid was associated with left ventricular hypertrophy (LVH), and hemodialysis patients with LVH had higher median pre-dialysis serum hippuric acid levels [81].

## 3. Free Water-Soluble Low-Molecular-Weight Uremic Toxins

Free water-soluble low-molecular-weight uremic toxins account for 46% of identified uremic toxins [12]. The upper molecular weight limit for water-soluble toxins is 500 Daltons (Da), and protein binding must be minimal. Water-soluble low-molecular-weight toxins tend to be among the uremic solutes with the highest fold change in kidney disease patients compared to healthy controls [12].

### 3.1. Trimethylamine N-Oxide

Trimethylamine N-oxide (TMAO) is a gut-derived free water-soluble low-molecular-weight uremic toxin [82]. Intestinal bacteria produce trimethylamine (TMA) from dietary choline, phosphatidylcholine, L-carnitine, and betaine. Conversion of choline to TMA occurs through TMA-lyase enzyme complex CutC/D [83], while L-carnitine and its derivative gamma-butyrobetaine are converted to TMA through the action of TMA-lyase enzyme complexes CntA/B [84] and YeaW/X [85]. TMA is subsequently converted to TMAO in the liver (Figure 1) by flavin-containing monooxygenases (FMO), namely FMO3 [86]. TMAO has been shown to accumulate in the plasma of CKD patients [87], and increased TMAO concentrations correlated with coronary atherosclerosis burden [21]. Patients with both type 2 diabetes and CKD were reported to have an increased abundance of TMA-producing gut bacteria compared to healthy patients [88]. TMAO is efficiently removed by dialytic clearance [87].

#### 3.1.1. Mechanisms for the Progression of CKD

Although the majority of research on TMAO has focused on its cardiovascular effects (as described in the next section), there is evidence that TMAO contributes to renal fibrosis. In a study of adult subjects undergoing coronary angiography, CKD patients with higher serum TMAO levels were found to be at higher risk for all-cause mortality [86]. In vivo studies have shown that a high-fat diet (HFD) or dietary supplementation of choline or TMAO promotes tubulointerstitial fibrosis and increases expression of pro-fibrotic genes and kidney injury markers [86,89]. Furthermore, HFD or supplementation with choline or TMAO increased phosphorylation of Smad3, suggesting an interaction of TMAO and the pro-fibrotic TGF-β1/Smad3 pathway [86,89]. Pharmacological inhibition of TMA production using choline analogues attenuated the detrimental effects of HFD [89] and choline/TMAO [90] and prevented renal dysfunction and fibrosis.

#### 3.1.2. Mechanisms for the Progression of CVD

Atherosclerosis is a chronic inflammatory disease with an autoimmune component in which the cells of the innate and adaptive immune systems are attracted into the atherosclerotic plaque [91]. TMAO has been implicated in this immune response observed in atherosclerosis. Dietary supplementation of either TMAO or choline to apoE-/- mice increased expression of the scavenger receptors SR-A1 and CD36 in peritoneal macrophages, increased macrophage recruitment to aortic lesions [92,93], and increased proatherogenic factors in the aortic arch [93]. The increased expression of CD36 and foam cell formation was mediated at least partly through the p38 MAPK and JNK1/2 pathway [93].

TMAO is also associated with the activation of the NLRP3 inflammasome, which has recently been implicated in the atherosclerotic process [94]. NLRP3 is a proteolytic complex composed of NLRP3, adapter protein ASC, and procaspase-1. When the inflammasome forms, procaspase-1 is cleaved to active caspase-1 and leads to the secretion of pro-inflammatory cytokines IL-1β and IL-18 through proteolytic cleavage of their respective precursors [94]. The activation of the NLRP3 inflammasome has been shown to induce inflammation and endothelial dysfunction. TMAO induced the formation and activation of the NLRP3 inflammasome and increased endothelial permeability. Disruption of the endothelial tight junction was prevented by silencing Nlrp3, suggesting this endothelial hyperpermeability was inflammasome-mediated [94]. ROS appears to play an important role in activating the NLRP3 inflammasome, as TMAO-induced mitochondrial ROS production was able to activate the NLRP3 inflammasome by decreasing the expression of sirtuin 3, a mitochondrial enzyme responsible for deacetylating and increasing the activity of superoxide dismutase 2 (SOD2) [95].

NF-κB signaling is also implicated in the atherogenic mechanisms of TMAO. TMAO has been shown to increase vascular inflammatory signaling [96] and promote leukocyte-endothelial cell adhesion [97], with NF-κB playing a role in both processes. Furthermore, activation of the NLRP3 inflammasome and NF-κB signaling by TMAO was recently reported to promote vascular calcification. Plasma levels of TMAO were found to be significantly higher in CKD patients with aortic arch calcification compared to patients without aortic arch calcification. TMAO induced calcification of VSMCs and upregulated expression of osteoblast-specific proteins in vitro and ex vivo. In vivo, administration of exogenous TMAO to nephrectomised mice exacerbated calcification compared to nephrectomy alone, while also increasing phosphorylated NF-κB and NLRP3 expression. Collectively, it was suggested that TMAO facilitates vascular calcification through both NF-κB signaling and NLRP3 inflammasome activation, and that these two mechanisms positively feedback with each other [98].

#### 3.1.3. Targets for Therapeutic Intervention-TMAO

TMAO is derived from dietary precursors and thus diet modulation has been investigated as a therapeutic intervention, with varying results. Choline and L-carnitine (TMAO precursors) are found in high abundance in red meat, eggs, and shellfish [99]. The effect of a Mediterranean diet on plasma TMAO levels has been investigated, with conflicting results. Urinary TMAO was reported to be significantly lower in vegetarian and vegan subjects compared to omnivorous subjects [100]. Conversely, in vascular patients, neither the intake of dietary precursors of TMAO nor Mediterranean diet adherence score were able to predict plasma levels of gut-derived metabolites, including TMAO [101]. A 6-month dietary intervention with the Mediterranean diet or the “Healthy Eating” diet (restriction on fat intake, and promotion of the consumption of fruits, vegetables, and whole grains) in healthy adult subjects also had no effect on fasting plasma TMAO levels [102]. Dietary intervention for the reduction of circulating TMAO remains a point of interest (NCT02016430, NCT03327805).

The use of probiotics to modulate the intestinal bacteria-mediated production of TMAO has also been investigated. Although pre-clinical experiments have demonstrated beneficial effects of probiotics in reducing plasma TMAO and protecting against atherosclerosis, [103,104], human clinical trials have reported varying results in the efficacy of probiotic supplementation. In a clinical trial of patients with cardiovascular risk factors, the administration of either lactofermented Annurca apple puree or *Lactobacillus* bacteria decreased plasma TMAO levels compared to baseline TMAO [105]. On the contrary, administration of a probiotic supplementation including lactobacilli and bifidobacteri to healthy subjects ingesting a high-fat, hypercaloric diet for 4 weeks had no effect on plasma TMAO concentrations [106]. Furthermore, three months of probiotic supplementation did not alter TMAO levels in either patients with metabolic syndrome or hemodialysis patients [107]. Clinical trials to assess the effects of probiotics, including assessing effects on plasma TMAO levels, are currently recruiting (NCT03418857, NCT03267758).

As previously mentioned, the synthesis of TMA from dietary precursors depends on the enzyme complexes CutC/D, CntA/B, and YeaW/X. As such, inhibition of these protein complexes may serve has a therapeutic target for reducing bacterial production of TMA. A choline analogue, 3,3-dimethyl-1-butanol (DMB) was demonstrated to inhibit CutC/D-mediated TMA synthesis and attenuate foam cell formation and atherosclerosis [108]. Based on the results from DMB, choline analogues iodomethylcholine (IMC) and fluoromethylcholine (FMC) were designed as non-lethal suicide substrate inhibitors for CutC. IMC and FMC both suppressed TMAO production, inhibited choline-induced platelet aggregation, and suppressed thrombus formation [109]. The gamma-butyrobetaine aza-analogue meldonium, a proposed inhibitor of CntA/B, also reduced the intestinal production of TMA and TMAO from L-carnitine, without affecting bacterial growth [110]. In a clinical study of 8 healthy subjects, the addition of meldonium to a TMA-rich diet decreased the diet-induced rise in plasma TMAO levels and increased urinary excretion of TMAO [111].

### 3.2. Asymmetric Dimethylarginine

Asymmetric dimethylarginine (ADMA) is a non-proteinogenic amino acid that is an endogenous competitive inhibitor of NOS and therefore NO production [112]. NOS uses L-arginine as a substrate to generate NO and L-citrulline. ADMA is synthesized through post-translational methylation of arginine in proteins by a family of protein arginine methyltransferases (PRMTs), specifically, type I PRMTs. Free ADMA is released upon proteolysis of ADMA-incorporated proteins. Circulating levels of ADMA are elevated in patients with CKD, even before alterations in GFR [113]. Approximately 20% of ADMA is excreted into the urine [112]. ADMA is primarily metabolized by dimethylaminohydrolase-1 (DDAH-1) and -2 (DDAH-2) [114], and alanine glyoxylate aminotransferase 2 (AGXT2) [115]. The kidneys play a central role in ADMA metabolism, as DDAH-1 and -2 are highly expressed in the kidneys and AGXT2 is found primarily in the kidneys [116]. The increased levels of ADMA observed in CKD are largely attributed to decreased activity of the metabolic enzymes of ADMA in CKD rather than decreased direct urinary excretion of ADMA [117]. 

#### 3.2.1. Mechanisms for the Progression of CKD

In a one-year follow up study of non-dialysis stage 3–5 CKD patients, elevated ADMA was shown to be a strong predictor of CKD progression for patients with baseline eGFR >36 mL/min/1.73 m^2^ [118]. While ADMA is implicated in renal fibrosis in CKD, whether it is fibrotic or anti-fibrotic is controversial. In vitro, ADMA was shown to increase cell proliferation, cell migration, and cell invasion of cultured rat kidney fibroblasts (NRK-49F) and mesangial cells, and increased the expression of fibrotic markers in rat kidney fibroblasts [119]. ADMA also induced ROS through upregulation of NOX4 expression, and it was proposed that ROS generated by NOX4 resulted in the phosphorylation of ERK1/2 and the activation of myofibroblasts [119]. In in vivo models of CKD, ADMA was associated with a decreased number of peritubular capillaries and increased renal fibrosis [120], and high levels of ADMA induced oxidative stress, expression of interstitial ECM proteins, and renal TGF-β1 expression [121]. Although these previous studies suggest a deleterious effect of ADMA in the progression of CKD, there are contradicting studies that indicate ADMA may have a protective effect against kidney damage. Proximal tubule specific knockout of Ddah-1 increased ADMA and was protective against the renal injury and fibrosis induced by both folate and unilateral ureteric obstructive (UUO) nephropathy [122]. Another study demonstrated that decreasing renal ADMA production by inhibiting type I PRMTs exacerbated renal fibrosis in UUO mice compared to control UUO mice. Interestingly, the addition of ADMA to NRK-49F cells treated with TGF-β reduced fibrosis, in contrast to the aforementioned study using the same cell line at an equal concentration of ADMA (100 μM) [119,123]. Evidently, there is much uncertainty to the role of ADMA with respect to renal fibrosis and kidney disease. It has been suggested that the effects of circulating ADMA and renal ADMA may have the opposite effects on fibrosis [122].

#### 3.2.2. Mechanisms for the Progression of CVD

Many studies have shown the association between circulating ADMA and CVD risk and mortality in a number of different populations. ADMA has been implicated with atherosclerosis, through the induction of oxidative stress and endothelial dysfunction. As previously mentioned, the most well-known effect of ADMA is inhibition of NO through competitive inhibition of eNOS. NO is well documented to be crucial in the maintenance of cardiovascular function, and impaired NO signaling has been shown to result in detrimental effects in both the vasculature and cardiac cells. ADMA has been shown to reduce NO synthesis both in vitro and in vivo [124], and vascular endothelial specific knockout of DDAH-1 in mice increased ADMA levels and reduced NO production in aortic rings [125]. ADMA was shown to drive ICAM-1 and VCAM-1 expression through the induction of NF-κB, and this ADMA-induced NF-κB signaling was regulated by p38 MAPK and ERK1/2 [126]. Long term ADMA treatment in mice induced superoxide production, medial thickening, and perivascular fibrosis in coronary microvessels, and increased angiotensin-converting enzyme (ACE) expression in the perivascular areas. Treatment with an ACE inhibitor and angiotensin receptor blocker prevented lesion formation and normalized ROS production, suggesting an interaction between ADMA and the RAS pathway [127]. ADMA was proposed to activate the RAS system, which leads to the activation of NADPH oxidases, further increasing ROS production and causing vascular dysfunction [128].

ADMA may additionally contribute to atherogenesis by promoting foam cell formation. ADMA induced the expression of scavenger receptor LOX-1 in HL-60 (human monocytic leukemia) cells, increased oxLDL uptake by more than 2-fold, and resulted in the accumulation of lipid droplets [129]. oxLDL decreased DDAH-2 expression in macrophages and increased intracellular ADMA levels, while ADMA potentiated lipid accumulation in the macrophages, suggesting a feedback effect between oxLDL and ADMA [129]. Additionally, ADMA reduced cholesterol efflux in macrophages, potentially by activating NADPH oxidases and increasing ROS. It was proposed that the increased ROS in the macrophages reduces liver X receptor alpha activity and consequently downregulates the expression of ABC transporters responsible for cholesterol efflux [130].

#### 3.2.3. Targets for Therapeutic Intervention-ADMA

The main therapeutic strategy currently being investigated for reducing circulating ADMA is increasing DDAH activity [131]. A number of in vivo studies have shown that increasing DDAH expression or activity reduces circulating ADMA and has beneficial effects in preserving endothelial function, preventing hypertension, and attenuating atherosclerosis [112]. The GW4064 (an FXR agonist), telmisartan, rosuvastatin and atorvastatin, and melatonin, among other compounds, were shown to either increase or prevent the decrease of DDAH activity/expression and exert cardioprotective effects [112]. Recently, quercetin, a dietary antioxidant, was shown to protect HUVECs against vascular damage caused by iron overload. It was determined that iron overload increased ADMA levels and decreased DDAH-2 activity/expression and NO production, all of which were reversed by quercetin. These protective effects of quercetin were negated by silencing DDAH-2, and it was proposed that quercetin likely reduced intracellular ROS, increasing DDAH-2 expression and preventing ADMA accumulation while increasing NO production [132].

L-arginine is the substrate for NOS in the production of NO. Lower L-arginine/ADMA ratio has been clinically associated with atherosclerosis, and thus increasing the L-arginine/ADMA ratio through supplementation with L-arginine or L-citrulline is another proposed therapeutic strategy against the effects of ADMA [112,133]. L-citrulline is converted into L-argininosuccinate by argininosuccinate synthetase, which can be subsequently converted back into L-arginine [134]. The addition of ADMA to isolated porcine coronary artery impaired vasodilation, downregulated eNOS expression and eNOS phosphorylation, decreased expression of argininosuccinate synthetase, reduced endothelial NO production, and increased superoxide production. Co-treatment of ADMA and L-citrulline significantly diminished the effects of ADMA, indicating that L-citrulline supplementation was able to preserve endothelial function [134].

Although a few recruiting or active clinical studies investigating the cardiovascular benefits of various compounds in kidney disease include the measurement of serum ADMA, the changes in ADMA levels are generally secondary or alternative outcomes in these investigations (NCT04635670, NCT03471117).

## 4. Middle Molecule Uremic Toxins

Middle molecules account for approximately 28% of currently identified uremic toxins [12]. Middle molecules have a molecular weight of over 500 Da and their size restricts clearance during hemodialysis [135]. There is evidence to support middle molecule contribution to morbidity and mortality in dialysis patients driving changes in dialysis technology to facilitate their clearance [135]. For example, cytokines are middle molecules that have received substantial interest as uremic toxins given their widely appreciated role in mediating inflammation, a key factor in CKD and CVD pathogenesis.

### 4.1. Tumor Necrosis Factor Alpha

Tumor necrosis factor alpha (TNF-α) is a proinflammatory cytokine produced by cells of the immune system such as activated T-lymphocytes, macrophages and mast cells, as well as other cells including vascular endothelial cells, tubular epithelial and mesangial cells of the kidney, and cardiomyocytes [136]. TNF-α exists in both soluble and transmembrane bound forms. Transmembrane TNF-α is presented as a cell surface type II polypeptide on activated T cells and macrophages and requires cleavage by TNF-α converting enzyme (TACE) into its 17 kDa soluble form, which can then bind receptors. Soluble TNF-α binds membrane bound TNF-α receptor type 1 (TNFR1) and TNF-α receptor type 2 (TNFR2), which are found on nearly all nucleated cells [137]. TNF-α levels in circulation are considered negligible or undetectable in the healthy state but increase in acute and chronic inflammation [136].

#### 4.1.1. Mechanisms for the Progression of CKD

CKD presents as a low grade, persistent, chronic inflammatory state. Research has focused on unravelling the complex role of innate and adaptive immunity in the sustained inflammatory milieu that ultimately leads to renal fibrosis and ESRD [138]. It has been documented that HD patients have elevated levels of TNF-α and IL-6, as they are not effectively cleared due to size restrictions of traditional dialytic membranes [139]. A relationship between TNF-α and renal function has been explored in both animal models and in humans with varying stages of CKD. In 75 CKD patients ranging from stage 2–5, it was demonstrated that plasma TNF-α levels were inversely related to GFR, beginning to accumulate at GFR <81 mL/min/1.73 m^2^ [140].

TNF-α is involved in the pathophysiology of hypertensive kidney disease and downstream renal damage [136]. Experimental evidence indicates that TNF-α contributes to renal vasoconstriction through an increase in superoxide generation, which reduces NO bioavailability, and leads to a subsequent reduction in GFR [141]. Binding of TNF-α to TNFR1 is reported to alter renal hemodynamics, reducing GFR and renal blood flow, while binding to TNFR2 signals for increased macrophage infiltration to the renal interstitium, ultimately leading to renal injury in the form of interstitial fibrosis and glomerulosclerosis [142]. This finding is in line with accumulating evidence that renal inflammation activates cells of the immune system, which plays a key role in pro-fibrotic mechanisms leading to ESRD [138].

#### 4.1.2. Mechanisms for the Progression of CVD

Both TNF-α and IL-6 have been identified as promoters of vascular calcification that can lead to phenotypic switching of VSMCs [143]. It has been recently discovered that phenotypical switching to osteoblastic VSMC and subsequent calcification is mediated through TNF-α [139]. Using human VSMCs and a uremic serum pool generated from samples of 14 patients undergoing HD, TNF-α was shown to induce IL-6 expression in VSMCs through the transcription factor AP-1 and proto-oncogene c-FOS pathway [139]. Blocking this signaling pathway almost entirely attenuated the calcification induced by uremic serum [139].

In addition to vascular calcification, TNF-α contributes to the progression of atherosclerosis at almost every stage of atherogenesis. In HUVECs, TNF-α stimulates expression of VCAM-1, ICAM-1 and E-selectin [144] by binding to TNFR1 and eliciting downstream NF-κB activation [144]. Expression of factors that promote interaction with the endothelium also enhance leukocyte rolling and firm adhesion [145], contributing to inflammation within the vascular wall in the formation of atherosclerotic plaques [146]. Indeed, a statistically significant relationship has been demonstrated between TNF-α and presence of carotid plaques in ESRD patients [147]. TNF-α is also capable of mediating blood lipid composition which is paramount in atherogenesis.

#### 4.1.3. Targets for Therapeutic Intervention—TNF-α

Generally targeting TNF-α would result in immune suppression, thereby potentially being more harmful, due to the attenuated ability to resolve acute infection [136]. However, modulation of TNF-α and other cytokine activity has yielded promising, and even successful, results in diseases such as psoriasis, rheumatoid arthritis and Crohn’s disease, by modulation of characteristically aberrant immune-inflammatory response [148]. Anti-TNF-α monoclonal antibody treatment (TNF-α blockers), have been clinically used in the treatment of the aforementioned diseases, which also carry a high risk of atherosclerosis and cardiovascular complications. In a study involving over 200 patients with psoriatic arthritis, half receiving traditional disease-modifying antirheumatic drugs (DMARDs) and half receiving a TNF-α blocker, patient carotid intima-media thickness (C-IMT) was measured as an indicator of subclinical atherosclerosis [149]. It was found that the C-IMT was significantly lower in patients on TNF-α blockers compared to those receiving traditional treatment [149]. However, not all TNF-α blockers have the same biologic activity, which leads to confounding factors when comparing their clinical effects, especially amongst heterogeneous patient populations [150].

A variety of other classes of drugs are also effective in limiting TNF-α production, beyond the direct neutralization activity of TNF-α blockers. ACE inhibitors are used in the treatment of kidney diseases to limit hypertensive renal injury, with much interest surrounding the immunomodulatory activity of captopril [151]. An in vivo experiment involving spontaneously hypertensive rats (SHR) demonstrated that higher mRNA and protein expression of inflammatory cytokines, such as TNF-α and IL-6, could be attenuated by captopril treatment [151]. Further, the group also observed higher phosphorylation of NF-κB and associated activation kinases in SHR than in controls, which could be suppressed by captopril treatment [151]. This demonstrates that the renoprotective effects of captopril may take place not only through lowering of blood pressure, but through modulation of pro-inflammatory gene transcription via NF-κB [151]. The association of ACE inhibitor treatment and lower plasma TNF-α levels has also been observed in ESRD patients [152].

Currently active or recruiting clinical trials that include measurement of TNF-α in the setting of CKD are mainly focused on studying the effects of pre/probiotics or dietary supplementation on cardiovascular/renal outcomes (i.e., NCT03228563, NCT03475017 NCT03689569).

### 4.2. Interleukin 6

IL-6, like TNF-α, is an inflammatory cytokine that plays a key role in both acute and chronic inflammation [153]. IL-6 binds the IL-6 α-receptor (IL-6R), which requires signal transducing β subunit glycoprotein 130 (gp130), together forming a heterodimeric signaling complex [154]. Membrane bound IL-6R (mIL-6R) is expressed on hepatocytes, megakaryocytes [154] and immune cells such as neutrophils, B and some T lymphocytes, and monocytes/macrophages [155], while gp130 is ubiquitously expressed [154]. IL-6 can also bind soluble IL-6R (sIL-6R), which then forms the required signaling complex with cell surface gp130, thereby allowing cells that do not express IL-6R to contribute to IL-6 signaling activity [154]. Binding of membrane bound IL-6R constitutes the classical pathway of IL-6 activation, which is involved in regulatory anti-inflammatory activity and hepatic acute phase response, whereas binding of sIL-6R represents IL-6 trans-signaling, which is responsible for the pro-inflammatory effects of IL-6 [154]. 

Synthesis of IL-6 can be induced by other cytokines also involved in inflammation, including TNF-α and IL-1α, as well as bacterial infection associated lipopolysaccharide, and viral infections [154]. These factors can activate NF-κB mediated transcription of the IL-6 gene, which is considered key for IL-6 expression; However, the IL-6 promoter region also contains binding motifs for AP-1, cAMP and CCAAT enhancer binding protein β, also known as NF-IL6 [154].

#### 4.2.1. Mechanisms for the Progression of CKD

IL-6 levels are commonly elevated in CKD patients [156], with a reported increase of 1.48-fold in the plasma of uremic patients [157]. In the analysis of mRNA expression in human CKD patient kidney biopsies, IL-6 was increased when compared with controls [158]. Although the involvement of IL-6 in the development and progression of renal interstitial fibrosis has been controversial, emerging experimental evidence points to a role of IL-6 trans-signaling [156]. In a study by Chen et al., it was shown that antagonism of the IL-6/sIL-6R complex by IgG Fc linked gp130 (Fc-gp130) decreased STAT3 phosphorylation and attenuated the development of renal fibrosis by interfering with IL-6 trans-signaling [156]. The authors addressed the discrepancy in the literature concerning the role of IL-6 in renal fibrosis, pointing to the inadequacy of comparing wildtype and IL-6 knockout mice, due to loss of classical IL-6 signaling which is responsible for anti-inflammatory activity in injured tissues [156]. Another compound associated with renal fibrosis is endothelin-1 (ET-1), which is a potent vasoconstrictor that has been implicated in hypertension [159]. Angiotensin II has been implicated in the induction of IL-6 expression through a variety of mechanisms and in a number of pathological settings [159]. PreproET-1 mRNA was significantly increased in the kidneys of angiotensin II infused C57BL/6 mice, and this upregulation was shown to be facilitated by IL-6 [159]. Inhibition or genetic deletion of IL-6 prevented the detrimental effects of angiotensin II, including hypertension, ET-1 expression, and renal injury/fibrosis [159]. The same study demonstrated that IL-6 was elevated in renal glomeruli and tubules of both hypertensive and normotensive CKD patients, although the extent of elevation was significantly higher in hypertensive CKD patients compared to normotensive CKD patients [159].

IL-6 trans-signaling has been suggested to be involved in expression of fibroblast growth factor 23 (FGF23), which has been well documented to not only be elevated in serum of CKD patients, but also strongly associated with mortality [160]. FGF23 is a key regulator of systemic phosphate homeostasis [161]. It has been demonstrated in vivo that IL-6 is necessary for increased expression of FGF23 in uremia [160]. FGF23 has been shown to bind hepatic FGF23 receptor 4, leading to increased expression of inflammatory cytokines such as IL-6, TNF-α and C reactive protein (CRP) [160], potentially indicating the role of a positive feedback loop between expression of IL-6 and FGF23. A cross-sectional analysis of FGF23 and markers of inflammation in the Chronic Renal Insufficiency Cohort (CRIC), a multicenter cohort comprised of nearly 4000 adult, Stage 2–4 CKD patients, has shown that FGF23 was independently associated with high levels of IL-6, fibrinogen, CRP and TNF-α, with correlation being strongest with IL-6 and fibrinogen [162]. Despite the strong correlation with inflammatory markers [162] and mortality [160], the prognostic relevance of FGF23 in patients with CKD is yet uncertain [161].

#### 4.2.2. Mechanisms for the Progression of CVD

IL-6 is considered a chief biomarker for cardiovascular risk in CKD with and without hypertension [163], and has been evaluated clinically in dialysis [164] and non-dialysis [165] patients. Specifically, investigation into prognostic ability of IL-6 has been of interest as a G/C polymorphism at the -174 position in the promotor region of the IL-6 gene, resulting in IL-6 overexpression, has been linked to all-cause mortality in peritoneal dialysis (PD) patients [164], as well as elevated risk of cardiovascular disease incidence [165]. A significant increase in serum IL-6 compared to baseline was associated with longer time receiving PD treatment and was predictive of cardiovascular events and suggestive of all-cause mortality [164].

IL-6 trans-signaling has been shown experimentally to mediate aldosterone induced cardiac fibrosis, which is supported by clinical evidence [166]. Aldosterone is well known to be released from the adrenal cortex and promote renal sodium retention and potassium excretion; However, aldosterone is also made elsewhere in the body, including the vasculature and myocardium [167], and participates in pathological roles that precipitate cardiovascular and renal injury [168]. Interference with the RAS system through ACE inhibition is common in the treatment of kidney disease, resulting in the decrease of aldosterone levels [167]. This effect is transient and often leads to a phenomenon termed “aldosterone breakthrough”, whereby aldosterone levels eventually increase in RAS antagonism [167]. Treatment of HUVECs with aldosterone has been shown to significantly increase IL-6 promoter activity, as well as IL-6 expression through mineralocorticoid receptor/PI3K/Akt/NF-κB signaling [166]. Additionally, cell culture experiments using human cardiac fibroblasts have demonstrated the role of IL-6 trans-signaling in the production of fibronectin and type I collagen after aldosterone treatment, as antagonism of sIL-6R with gp130 suppressed both mRNA and protein levels of fibrotic elements [166]. These results were confirmed in a mouse model, as continuous aldosterone infusion significantly increased cardiac interstitial and perivascular fibrosis, and these manifestations were rescued by antagonism of mineralocorticoid receptors and sIL-6R [166]. 

Elevated FGF23 in CKD has been independently associated with increased left ventricular mass index and LVH [169]. It has been demonstrated that FGF23 is directly capable of inducing pro-hypertrophic factors in isolated neonatal rat ventricular cardiomyocytes through binding of FGF receptor, and activation of associated calcineurin-NFAT signaling cascade [170]. The ability of FGF23 to promote hypertrophic growth of cultured cells was further confirmed in a mouse model, where injection of FGF23 lead to development of LVH [170]. The association of FGF23, IL-6 and LVH has also been identified in a cohort of 62 CKD patients receiving continuous ambulatory peritoneal dialysis [171], where circulating levels of FGF23 and IL-6 were most significantly increased in patients with LVH [171].

#### 4.2.3. Targets for Therapeutic Intervention—IL-6

Tocilizumab is a humanized anti-IL6R monoclonal antibody belonging to the IgG1 class that has shown considerable success in the treatment of chronic inflammatory disease [172], but has not previously been used in the treatment of CKD or associated cardiovascular complications. Tocilizumab mediates the IL-6 signal cascade by preventing binding to both soluble and transmembrane forms of IL-6R, and thereby disrupting trans- and classical IL-6 signaling, respectively [172]. Use of tocilizumab has been approved for the treatment of rheumatoid arthritis in over 100 countries, and is considered a safe and well tolerated drug in this patient population [172]. A significant body of literature exists for the success of tocilizumab in off-label uses in the treatment of a number of immune mediated and chronic inflammatory diseases, such as Crohn’s disease, atherosclerosis and type 2 diabetes mellitus [172], which may suggest a place for tocilizumab in the treatment of CKD. However, as mentioned earlier, IL-6 signaling through membrane bound IL-6R mediates anti-inflammatory and regenerative processes after wounding, thus global blockade of IL-6R may attenuate these effects [173]. Soluble gp130 (sgp120) acts as an endogenous inhibitor of sIL-6R and trans-signaling, thereby holding therapeutic potential [173]. Antagonism of IL-6 trans-signaling through administration of Fc-sgp130 protein has shown promising results in animal models of inflammatory bowel disease and colon cancer [173]. Therefore, direct neutralization of IL-6R and cofactor gp130 may offer therapeutic potential in CKD, however, questions remain into how effective these prospective treatments may be in downstream IL-6 effects, such as FGF23 and aldosterone attenuation.

There is widespread interest in the removal or inhibition of IL-6, and many clinical trials are investigating or are planning to investigate the effects of therapeutic interventions on IL-6 levels. For example, recruitment is currently ongoing for a phase 2 clinical trial evaluating the anti-inflammatory effects of an anti-IL-6 monoclonal antibody, ziltivekimab, in CKD patients (NCT04626505).

## 5. Conclusions

Many uremic toxins have been identified, but there are many more that are likely yet to be discovered. Out of the 100+ uremic toxins that are known, only a few have been extensively studied. Investigations of uremic toxins have generally been investigated independently, despite the fact that many toxins are elevated in the setting of CKD and likely work together in the progression of CKD and CVD. This review illustrates the point that uremic toxins modulate many of the same pathways to mediate toxicity and may therefore have additive effects. Both the individual and combined pathophysiological mechanisms of uremic toxins must be studied further for a better understanding of their roles in the progression of CKD and CVD. Additionally, highlighted in this review are potential targets for therapeutic intervention against the toxic effects of uremic solutes, many of which focus on reducing serum levels of uremic toxins. Further investigation is required to assess the benefit of currently known therapeutic targets and to identify additional novel therapeutic targets for the treatment of CKD and CVD. However, it is important to note that uremic toxins are not the only driver of disease progression in CKD and CVD, and many other factors contribute to disease progression [174]. Thus, targeting uremic toxins alone may be insufficient in slowing down or treating disease outcomes. For example, other non-uremic toxin related therapeutic options exist for mitigating CVD in CKD, such as anticoagulant, antiplatelet or lipid lowering therapies to reduce the risk of cardiovascular disease in CKD patients [175]. Perhaps a combined approach of targeting uremic toxins and managing the other aspects of disease progression is necessary for treating CKD and CVD.

## Figures and Tables

**Table 1:** Summary of uremic toxin properties, toxicity, and therapeutic interventions.

Molecule	Size (MW)	Protein Binding	Dialyzability	Fold Change (M/N) *	Origin	Site of Toxicity	Mechanism of Toxicity	Therapeutic Interventions
Indoxyl Sulfate	213.2	93% bound to albumin	32% cleared through dialysis	43.2 [12]	Metabolism of tryptophan by colon microbes.	Kidney proximal tubule cells, cardiomyocytes, endothelial cells, and VSMCs.	Generation of ROS, induction of fibrosis/inflammation in kidneys. Induce oxidative stress in VSMC.	AST-120. Pre-, pro-, and synbiotics. Dietary modulation.
p-Cresyl Sulfate	188.2	90% bound to albumin	29% cleared through dialysis	11.0 [12]	Metabolism of aromatic amino acids by colon microbes.	Kidney proximal tubule cells and endothelial cells.	Generation of ROS, induction of fibrosis/inflammation in kidneys and endothelial cells.	AST-120. Pre-, pro-, and synbiotics. Dietary modulation.
Hippuric Acid	179.2	34–40% bound to albumin	64% cleared through dialysis	23.8 [12]	Metabolism of dietary polyphenols by colon microbes.	Renal tubular cells and endothelial cells.	Generation of ROS, promotes renal fibrosis and endothelial dysfunction.	Potential interventions: Pre-, pro-, and synbiotics. Dietary modulation.
Trimethylamine N-oxide (TMAO)	75.1	Free water soluble	85% cleared though dialysis	28.6 [21]	Metabolism of dietary precursors choline, phosphatidylcholine, L-carnitine, and betaine by colon microbes.	Renal tubular cells, endothelial cells and VSMCs.	Induction of renal fibrosis. Enhance immune response in atherosclerosis.	Diet modulation. Probiotics. Choline analogues such as DMB, IMC and FMC.
Asymmetric Dimethylarginine (ADMA)	202.3	30% bound to albumin [22]	20–40% cleared through dialysis [23]	>6.4 [12]	Non-proteinogenic amino acid synthesized through post translational methylation of arginine by PRMTs.	Renal tubular cells, vasculature, and cardiomyocytes.	Renal fibrosis, generation of ROSInhibitor of NOS leading to impaired NO signaling. Promote foam cell formation.	Potentiate ADMA metabolism by increasing DDAH activity. Dietary antioxidants (i.e., Quercetin). L-arginine supplementation.
Tumor Necrosis Factor alpha (TNF α)	17,300	N/A	Minimal	3.09 [12]	Largely from immune cells (T lymphocytes, macrophages, mast cells), and vascular endothelial cells, renal tubular epithelial and mesangial cells, cardiomyocytes.	Renal proximal tubule, glomerulus and interstitium, vasculature.	Fibrosis, glomerulosclerosis, superoxide generation, macrophage infiltration, vascular calcification, atherosclerosis.	TNF- α blockers (e.g., Adalimumab, etanercept, infliximab). ACE inhibitors (e.g., Captopril).
Interleukin- 6 (IL- 6)	21,000	N/A	Minimal	1.48 [12]	Hepatocytes, megakaryocytes, immune cells (neutrophils, B- and some T-cells, monocytes/macrophages).	Renal tubules, glomerulus, interstitium, cardiac fibroblasts and myocytes, vasculature	Renal fibrosis, cardiac fibrosis (left ventricular hypertrophy), atherosclerosis.	Neutralization of soluble and membrane bound IL-6 receptors (e.g., Tocilizumab) and gp130 (e.g., Bazedoxifene).

* Concentration fold change (M/N) calculated by comparing the average concentration in adult hemodialysis patients (M) with the normal concentration (N) measured in healthy controls.
